# Mouse models of cerebral injury and cognitive impairment in hypertension

**DOI:** 10.3389/fnagi.2023.1199612

**Published:** 2023-07-19

**Authors:** Marialuisa Perrotta, Daniela Carnevale, Lorenzo Carnevale

**Affiliations:** ^1^Department of Molecular Medicine, “Sapienza” University of Rome, Rome, Italy; ^2^Research Unit of Neuro and Cardiovascular Pathophysiology, IRCCS Neuromed, Department of Angiocardioneurology and Translational Medicine, Pozzilli, Italy

**Keywords:** hypertension, cognitive impairment, cerebral injury, experimental murine models, vascular dementia

## Abstract

Hypertension is a major risk factor for dementia, including both vascular and neurodegenerative etiologies. With the original aim of studying the effect of blood pressure elevation on canonical target organs of hypertension as the heart, the vasculature or the kidneys, several experimental models of hypertension have sprouted during the years. With the more recent interest of understanding the cerebral injury burden caused by hypertension, it is worth understanding how the main models of hypertension or localized cerebral hypertension stand in the field of hypertension-induced cerebral injury and cognitive impairment. With this review we will report main genetic, pharmacological and surgical models of cognitive impairment induced by hypertension, summarizing how each specific category and model can improve our understanding of the complex phenomenon of cognitive loss of vascular etiology.

## 1. Introduction

Hypertension is globally recognized as one of the main and most diffused risk factors for the development of Alzheimer’s Disease (AD) and various forms of dementia (Gorelick et al., 2011; Iadecola et al., 2016). However, the research on the pathological consequences of hypertension has been mainly aimed at understanding its detrimental effect on the canonical organs affected by this condition, namely, the heart, the vasculature and the kidneys (Chobanian, 2009; Coffman, 2011). On this notice, the basic science studies that dissected the molecular and cellular mechanisms underlying blood pressure increase extensively focused on the alteration of signaling pathways modulating peripheral organs’ function (Coffman, 2011; Perrotta et al., 2016; Drummond et al., 2019), with the brain and its regulatory system studied for its role in controlling the autonomic nervous system, the vascular tone and the baroreflex (Esler, 2011; Carnevale and Lembo, 2021). The opposite route, i.e., how blood pressure impact brain structure and function in the long term, has been object of more recent investigation (Santisteban et al., 2023). Thus, both clinical and preclinical investigations in the effect of blood pressure on cognition and the mechanism by which it damages the brain have a more recent history, compared with the investigation on cardiac and vascular injury. Specifically, several works on hypertensive patients shown that the brain is indeed injured by hypertension (Maillard et al., 2012; Carnevale et al., 2018, 2020; Siedlinski et al., 2023), and recent trials have shown that an intensive treatment of blood pressure levels may not be a sufficient strategy to prevent dementia (Williamson et al., 2019). This suggests that hypertensive brain injury goes beyond a mechanical model, whereby the elevated blood pressure has a direct effect on the cerebral vessels and pushes toward the search for the molecular mechanisms driving the hypertensive cerebral injury and resulting in cognitive impairment.

On the other hand, AD and dementia research has mainly focused on countering the principal hallmarks of these diseases, like the β-Amyloid (Aβ) accumulation, the neurofibrillary tangles, the loss of neurons and gray matter atrophy. To investigate the mechanisms driving these phenotypes, the scientific community has produced and investigated many genetic models aimed at reproducing in mouse the alterations found in the forms of familial AD (Watamura et al., 2022). However, these models recapitulate only a part of the dementia spectrum and do not consider the vascular component of the sporadic AD, often underestimated also in the clinical practice, with evidences suggesting that almost half of the clinically diagnosed AD have a mixed vascular etiology (Santisteban and Iadecola, 2018). While this have been overseen for a long time, in the last 20 years the scientific and medical community has produced a growing body of literature underlining the importance of the vascular etiology in the dementias, thus calling for experimental models of cerebral vascular injury to investigate the mechanisms through which hypertension exerts damage to the brain and in turn cognition.

Starting from experimental models of hypertension, many studies in the last years have investigated different components of the cognitive impairment induced by vascular damage. The complexity of the hypertensive pathology, intrinsically developed in multiple compartments of the body and due to the interaction of different systems among them, resulted in a wide range of models in which hypertension, or localized forms of cerebral hypertension, are induced and developed with various strategies, like genetic, pharmacological, and surgical models (Lerman et al., 2019).

## 2. Genetic models of hypertension with associated cognitive impairment

Genetic models of hypertension and vascular injury were obtained by either genetic selection or genetic engineering of single genes involved in blood pressure regulation or vascular damage. These models have given the scientific community interesting insights, spanning from the complex manifestation of cerebral injury in spontaneous hypertensive mice to the fine regulations of the cerebrovascular tree fitness operated by specific genes as hypoxia-inducible factor 1 (HIF-1) or specific neurotransmitter receptors. Finally, manipulating the renin-angiotensin system (RAS) let us evaluate the effect of human renin ad angiotensinogen overexpression in a mouse model, thus permitting the investigation of the mechanisms solicited by this pathway responsible for blood pressure increase in a fraction of hypertensive patients.

### 2.1. BPH/2

BPH/2 mice have been generated by selection for hypertensive phenotype from the Jackson Lab (Schlager and Sides, 1997). These mice have been characterized for a specific pattern of cognitive impairment which strictly resembles the phenotype commonly observed in hypertensive patients (Fuemmeler et al., 2011; Hartman et al., 2012). These mice shown an hyperactive behavioral pattern, impaired spatial learning and spatial memory, with a further cognitive deficit in the cued learning domain and motor domain specific for the males (Hartman et al., 2012), investigated by water maze, rotarod test and open field activity test. This is a typical model of neurogenic hypertension, in which the GABA neurotransmitter modulation drives a sympathetic overactivity in the hypothalamic regions (Jackson et al., 2019). Leveraging this model it has been demonstrated the pivotal role of the cerebral innate immune cells, specifically cerebral perivascular macrophages (Faraco et al., 2016b), which enhance the Reactive Oxygen Species (ROS) production in the brain after being activated from Angiotensin-II (AngII). BPH/2 mice undergoing a depletion of this specific macrophagic population showed a rescue of the cognitive impairment phenotype in the specific domains of recognition and spatial memory evaluated, respectively by novel object recognition test and Barnes maze test.

### 2.2. Renin/Angiotensinogen

The manipulation of RAS has generated a model of spontaneous hypertension in which crossing a transgenic mouse overexpressing human Renin (hRen) and a mouse overexpressing human Angiotensinogen. The resulting breed shows a marked increase in systolic blood pressure, and interestingly vascular remodeling of cerebral vessels dependent on the RAS (Baumbach et al., 2003), and a cognitive impairment characterized with the shuttle avoidance test dependent on brain angiotensin signaling and not on the SBP levels (Inaba et al., 2009).

### 2.3. Other genetic models leading to cerebral injury and dementia of vascular etiology

Further investigations in transgenic mice have either individuated genes responsible for cerebral injury due to vascular etiology or genes whose absence exacerbate the cerebral damage. As an example, according to recent reports mice lacking the myosin phosphatase target subunit 1 (a regulator of smooth muscle cells contraction) in the smooth muscle cell are spontaneously hypertensive and exhibit the main characteristics of cerebral small vessel disease (cSVD) and cognitive impairment (Chen et al., 2023). Mice lacking the GABA receptor β3 subunit in the endothelium show cognitive impairment of the spatial domain observed by the spatial novelty Y maze, alterations in cortical blood flow and cardiac and vascular remodeling (Agrud et al., 2022). Finally, mice with a specific depletion of HIF-1 in the pericytes shown decreased sensibility to hypoxia induced cerebral vascular remodeling and increased vascular permeability (Baumann et al., 2022). Endothelial cells, smooth muscle cells and pericytes, astrocytes and neurons form the neurovascular unit, an ensemble of interplaying cells regulating the vascular-neuronal homeostasis and physiological activity. Taken together, these finding suggest that the complex interplay played in this unit is a sensible mechanism which can be disrupted by several challenges targeting its components, and thus a deep understanding of its behavior in pathophysiological contexts may provide important insights for new therapeutical targets in cerebrovascular diseases.

## 3. Pharmacological models of hypertension with phenotype of cerebral injury and cognitive impairment

Pharmacological induction of hypertension is a repeatable way to induce vascular and cerebral injury in mice targeting the main culprits of the clinical manifestation of hypertension, like AngII or ROS. Furthermore, it is also possible to leverage models that mimic specific forms of hypertension as salt-sensitive hypertension obtained by administration of Deoxycorticosterone-acetate (DOCA)-salt tablets. The possibility to reproduce these models across a big variety of laboratories makes the findings obtained very stable and reproducible, thus making them good candidates for the study of hypertension-induced cognitive impairment and cerebral injury.

### 3.1. Angiotensin-II infusion

AngII infusion is one of the main experimental models of hypertension, and its effects have been described in a large literature in many districts, spanning from local and tissue-specific effects to systemic effects (Crowley et al., 2006; Guzik et al., 2007; Okuno et al., 2023). The strong evidence that blocking the action of the RAS has positive effects on hypertension and its associated organ damage resulted in the adoption of RAS targeting drug as the primary choice for hypertension treatment (Whelton et al., 2018; Williams et al., 2018). What is still under investigation and debatable is whether RAS targeting prevents cognitive impairment and cerebral damage induced by hypertension. In this context, AngII-dependent hypertension is a powerful model to investigate this question. AngII infusion in mice results in a marked cognitive impairment and behavioral alterations (Duchemin et al., 2013; Gao et al., 2021) in the domains of learning and memory. AngII infused mice show a series of correlated dysfunctions, ranging from the alteration of cerebrovascular autoregulation (Toth et al., 2013), to neurovascular dysfunction (Faraco et al., 2016b) and an exacerbation of the adverse cerebral effects observed in a genetic model of AD (Faraco et al., 2016a). Several findings suggest that the action of AngII on cognitive impairment may be independent from BP levels (Faraco et al., 2016b; Kerkhofs et al., 2020), and thus treating hypertension with the sole aim of lowering the BP levels and mechanical stress to cerebral vasculature may be an inadequate solution for hypertension-induced cognitive impairment.

### 3.2. Deoxycorticosterone-acetate salt

Deoxycorticosterone-acetate -salt administration is an experimental model mimicking the salt-sensitive hypertension exhibited by a portion of the hypertensive patients which respond to high salt intake with an elevation of BP levels (Basting and Lazartigues, 2017). It has been demonstrated that this model, despite reproducing hypertension with low peripheral RAS activity and low circulating AngII levels (Gavras et al., 1975), results in an elevation of RAS cerebral activity (Grobe et al., 2010), neuroimmune axis recruitment (Perrotta et al., 2018), and cerebral damage (Rodrigues and Granger, 2012). In this light, this model is a potent tool to explore the cerebral effects of RAS activation without elevated circulating levels of AngII obtained by the peptide infusion. Additionally, DOCA-salt model demonstrated that the cerebrovascular damage in hypertension is indeed mediated by a cerebral activation of the AngII receptor, and that RAS blocking strategies that exert their effect in the brain are able to hamper this process (Rodrigues and Granger, 2012). Interestingly, while the cerebral injury following DOCA-salt administration have been thoroughly characterized in mice, no study investigated in which way these cerebral alterations may impact cognition.

### 3.3. L-NAME

The role of Nitric Oxid Synthase (NOS) and ROS have always been investigated in hypertension and in the correlated organ damage. The manipulation of this balance can be altered in simple yet effective way, like inhibiting the NOS by administering L-NAME (Küng et al., 1995). The role of NO in cognition and cognitive impairment has been demonstrated even in non-human primates (Prendergast et al., 1997) and then investigated in small animals (Hendrickx et al., 2021) by the Morris water maze, suggesting its importance in a translational perspective.

## 4. Mechanical and other models of blood pressure alterations inducing cognitive impairment

The induction of cerebral injury and hypertension-like injury in the brain can be obtained by procedures which do not alter either the genetic or hormonal balance of the animal in a direct way, leveraging external stimuli or surgical procedures to obtain this. The examples of transverse aortic constriction or bilateral carotid artery stenosis are very relevant to this. In fact, they impose a cerebrovascular stimulus with a direct mechanical effect on the vascular district directed to the brain, imposing in a fast and reproducible way a localized cerebral hypoperfusion like the one induced by chronic arterial hypertension, without inducing systemic hypertension. On the other hand, hypoxia models are useful to represent the important risk factor of obstructive sleep apnea, where a mechanical obstruction of the airways during sleep elicits an important hypertensive stimulus and is able to exacerbate *per se* the cerebral and cognitive injury. It is worth noting that the possibility to reproduce the cerebral effects of hypertension with mechanical procedures as TAC or BCAS, without altering systemic blood pressure levels is a potent tool for the researcher to be able to separate the study of the mechanical effects of elevated blood pressure on cerebral vessels from the effects of its most common consequences on the brain, as hypoperfusion and neuroinflammation.

### 4.1. Transverse aortic constriction (TAC)

Transverse aortic constriction (TAC) is a surgical procedure to induce a constriction of the aortic arch between the anonymous trunk and the carotid artery. The model was originally developed to investigate the consequences of cardiac pressure overload (Rockman et al., 1991), however the imbalance between left and right carotid blood flow develops a severe form of cerebral hypertension, without affecting systemic BP (Poulet et al., 2006). This characteristic made the model an interesting tool to investigate the brain injury in a controllable hypertension model. By leveraging this model, it has been demonstrated the presence of Aβ plaques and neuroinflammation in brain sustaining hypertensive injury (Carnevale et al., 2012a), the subsequent cognitive impairment (Carnevale et al., 2012b) dependent on the RAGE activation in cerebral vasculature and the mechanism of cerebral and cognitive injury when imposed on models prone to genetic forms of cognitive impairment (de Montgolfier et al., 2019a) or the additive effect in metabolic pathologies inducing cognitive impairment (de Montgolfier et al., 2019b). Finally, investigating the immune contribution to the cognitive impairment and microvascular damage in this model revealed a pivotal role of inflammatory cytokines as interferon-γ (Apaydin et al., 2022), exerting its role on resident innate immunity of the brain and leading to microvascular rarefaction and cognitive impairment. The TAC impacts mainly the spatial and learning domain, assessed with Morris water maze (Carnevale et al., 2012b; de Montgolfier et al., 2019b; Apaydin et al., 2022) and Novel object recognition (Carnevale et al., 2012b).

### 4.2. Bilateral carotid artery stenosis (BCAS)

In the last 20 years another surgical model of cerebral hypoperfusion has been developed and extensively used, namely, the Bilateral Carotid Artery Stenosis (BCAS) (Shibata et al., 2004). This model is obtained by applying dual coils to the carotid arteries in order to reduce the cerebral blood flow, resulting in white matter injury and neuroinflammation, thus being a good model for clinical hypertension-induced cerebral injury. This pattern of damage has been demonstrated to be accompanied in a loss of cognitive function (Nishio et al., 2010) with MRI imaging markers of damage similar to the one obtained in hypertensive patients (Hall et al., 2022). Several studies demonstrated that also in this model the interplay between cerebral hypoperfusion, vascular damage and cognition is mediated by a neuroinflammation (Mogi et al., 2018; Poh et al., 2021), adaptive (Wang et al., 2022) and innate cerebral immunity (Kakae et al., 2019). Finally, the alterations induced by this model in the brain glymphatic functions have been investigated to understand the potential mechanisms underlying the additional risk imposed by cerebrovascular factors in AD, proposing the reduced glymphatic drainage and reduced vascular pulsation may be key in the accumulation of β-amyloid amyloid in the brain (Li et al., 2021). The BCAS model has been shown to induce cognitive impairment in the domains of working memory, spatial memory and learning, and has been extensively characterized at several time points ranging from 14 days to 8 months of stenosis.

### 4.3. Hypoxia

The obstructive sleep apnea (OSA) is an independent risk factor for dementias and also a driver of hypertension. Thus, it is interesting studying how this diffused clinical condition might interact with blood pressure levels to develop cognitive impairment, and which are the common and independent mechanisms by which it happens. To this aim, models of chronic intermittent hypoxia have been developed in murine models by exposing mice to air with reduced oxygen content in a cyclic way for several hours per day. By this way, it has been demonstrated that endothelial dysfunction affects neurovascular coupling (Capone et al., 2012), induced cerebral neuroinflammation (Sapin et al., 2015), and cognitive dysfunction (Shen et al., 2021) in the domains of spatial memory and learning. It has also been shown that OSA, when combined with sleep fragmentation, exacerbates the cognitive damage and neuroinflammation in a model of familial AD (Qian et al., 2022), underlining its potential contribution both as independent and combined risk factors in various forms of dementia and cognitive impairment.

## 5. Discussion

Hypertension is a multifactorial risk factor, is influenced by several genetic, lifestyle and environmental factors and in the clinical practice there is the need to personalize the therapy to understand which kind of treatment is best suited to control both blood pressure levels and target organ damage in every single patient (Sever, 1995). While research on forms of dementia with neurodegenerative etiology has focused on transgenic models summing up all the identified genetic variants (Watamura et al., 2022), the heterogeneity of models developed to recapitulate the complex paradigm of cerebrovascular injury in hypertension is a powerful added value, since the different stimuli can shed light on the multifaceted processes that lead to cerebral and cognitive injury (Table 1). On this, animal models in general demonstrated to be fundamental to investigate the different organ damage and pathological mechanisms involved and recruited in hypertension (Lerman et al., 2019) and for brain injury (Obari et al., 2016). However, in this review we focused on mouse models as they proven as the most versatile models for the widely available genetic tools that let us mechanistically intervene and modify the pathological processes affecting cognition.

**TABLE 1:** Models of hypertension induced cerebrovascular injury or cognitive impairment with investigated phenotypes and relevant literature references.

Model	Cerebrovascular injury	Tests for cognitive impairment	References
*Genetic models of hypertension-induced cerebrovascular injury or cognitive impairment*			
***BPH/2 mice*** *Spontaneously hypertensive*	● Cerebrovascular oxidative stress ● Neurovascular dysfunction	Novel Object Recognition Test, Barnes Maze Test	Faraco et al., 2016b
***Double transgenic hRN/hANG mice*** *Overexpression of both human renin (R +) and human angiotensinogen (A +)*	● Continuous activation of the human brain renin- and angiotensinogen	Shuttle Avoidance	Inaba et al., 2009
***MYPT1^**SMKO**^ mice*** *Knockout for the specific myosin phosphatase target subunit 1 (MYPT1) on vascular smooth muscle cell.*	● Cerebral small vessel disease-like small vessel impairment.	Y Maze Test, Elevated Plus Maze Test, Morris Water Maze Test	Chen et al., 2023
***Gabrb3^**ECKO**^ mice*** *Conditional knockout for GABA_*A*_ receptor β3 subunit endothelial cell.*	● Reduction in vessel densities	Y Maze Test	Agrud et al., 2022
***GFAP-CreER^**T2**^; HIF-1α^**fl**/**fl**^ mice and GLAST-CreER^**T2**^; HIF-1α^**fl**/**fl**^ mice*** *Deletion of HIF-1 gene by recombination with Cre-inducible in astrocyte*	● Hypoxia-induced vascular remodeling	Not investigated	Baumann et al., 2022
***Pharmacological models of hypertension-induced cerebrovascular injury or cognitive impairment***			
***Angiotensin II (AngII)-hypertensive mice*** *IP infusion of AngII*	● Neural oscillations via altering excitatory and inhibitory (E/I) balance	Novel Object Recognition Test, Morris Water Maze Test	Gao et al., 2021
***AngII-hypertensive mice*** *AngII infusion (1,900 ng/kg/min) by osmotic minipumps for 14–21 days*	● Increased cerebral reactive oxygen species production ● Neurovascular coupling impairment	Morris Water Maze Test	(Duchemin et al., 2013)
***AngII-hypertensive mice*** *AngII infusion (1,000 ng/min per kg) by osmotic minipumps for 28 days*	● Alteration of cerebrovascular autoregulation ● Microvasculature rarefaction ● Neuroinflammation	Elevated Plus Maze Test	Toth et al., 2013
***AngII and DOCA - salt in in Tg2576 mice*** *(subcutaneously, 600 ng/kg of AngII per minute for 14 days or 50 mg DOCA pellets–21 days release*	● Microvascular amyloid deposition ● Enhanced β-secretase APP cleavage	Not investigated	Faraco et al., 2016a
***AngII-hypertensive mice*** *AngII infusion (1 μg/kg/min) by osmotic minipumps for 12 weeks*	● Microglia depletion ● BBB leakages	Morris Water Maze Test, Object Location Task	Faraco et al., 2016a
**DOCA - salt hypertensive mice** subcutaneous DOCA pellet-50 mg, 21-day release	● Neuroinflammation	Not investigated	Rodrigues and Granger, 2012
***N-Nitro-L-arginine methylester (L-NAME) hypertensive mice*** *L-NAME administration (0.5 mg/mL) in drinking water for 2-8-16 weeks*	● NO dysfunction	Morris Water Maze Test	Hendrickx et al., 2021
***Mechanical and other models of cerebrovascular injury or cognitive impairment***			
***Transverse Aortic Constriction (TAC)*** *after 1 to 7 days*	● Brain hypoperfusion Oxidative stress in the brain ● Neuroinflammation	Not investigated	Poulet et al., 2006
**TAC** after 3 weeks	● Brain hypoperfusion ● Early neuroinflammation ● Late-Brain Aβ Deposition	Novel Object Recognition Test, Morris Water Maze Test	Carnevale et al., 2012a,b
***TAC*** *after 6 weeks*	● Brain hypoperfusion Microhemorrhages ● Loss of microvascular density ● Brain atrophy	Morris Water Maze Test	de Montgolfier et al., 2019a,b
***TAC*** *after 4 weeks*	● Cortical capillaries rarefaction	Morris Water Maze Test	Apaydin et al., 2022
***Bilateral Carotid Artery Stenosis (BCAS)*** *after 3, 7, 14, or 30 days*	● Gray Matter change ● Proliferation of Activated microglia and astroglia	Not investigated	Shibata et al., 2004
***BCAS*** *from 6 to 8 months*	Hippocampal atrophy	Working Memory test	Nishio et al., 2010
***BCAS*** *from 6 to 8 months*	Small vessel disease	Novel Object Recognition Test, Morris Water Maze Test	Hall et al., 2022
**BCAS** after 6 weeks	Decreased cerebral blood flow	Morris Water Maze Test	Mogi et al., 2018
**BCAS** after 32-34 days	● Decreased cerebral blood flow ● Activation of inflammasome components and their downstream products (IL-1β and IL-18)	Morris Water Maze Test	Poh et al., 2021
**BCAS** after 14 days and 28 days	White Matter Damage	Novel Object Recognition Test Morris Water Maze Test	Wang et al., 2022
***BCAS*** *after 28 days*	● White Matter Damage ● Inflammatory responses in the corpus callosum	Novel Object Recognition Test	Kakae et al., 2019
***BCAS*** *after 3 months*	● Neuro-glial-vascular unit dysfunction ● Accumulation of Aβ protein ● Impaired glymphatic drainage ● Reduced vascular pulsation	Barnes Maze Test	Li et al., 2021
***Intermittent hypoxia*** *for 14 or 35 days*	● Neurovascular dysfunction ● Oxidative stress in cerebral resistance arterioles	Not investigated	Capone et al., 2012
***Intermittent hypoxia*** *for 1 day, 6 or 24 weeks*	● Chronic Low-Grade neuroinflammation in dorsal hippocampus	Not investigated	Sapin et al., 2015
***Intermittent hypoxia*** *for 2 or 4 weeks*	● Apoptotic bodies in the ● Changes in p-CREB and BDNF	Novel Object Recognition Test, Morris Water Maze Test	Shen et al., 2021
***Intermittent hypoxia*** *and sleep deprivation*	● Cholinergic neurodegeneration	Novel Object Recognition Test, Y maze Test, Active and passive place avoidance Test, Morris Water Maze Test	Qian et al., 2022

While all models contribute to brain injury with different pathogenic mechanisms and recruiting different molecular pathways, they often converge on a restricted number of fundamental processes affecting cognition, like the neurovascular unit efficacy, the neuroinflammation preceding and driving the cerebral injury and the pivotal role of innate cerebral immunity. Thus, the crossed comparison of cognitive impairment phenotype and molecular pathway involved in different models of cerebral hypertension may be an important field of research in the upcoming years, to fully characterize which are the fundamental mechanisms driving cognitive impairment in the clinical manifestation of the pathology and, most importantly, how and whether they are able to sum up their effects and drive a progressive detrimental process on the cognition, bridging from mild forms of cognitive impairment to severe dementia or neurodegenerative pathologies as Alzheimer’s Disease. One limitation of this review is the lack of detailed discussion of emerging mechanisms potentially affecting the cognition and the cerebral vasculature, like glymphatic clearance, that have not been characterized in several of the models discussed, and thus are still matter of ongoing investigations and debate.

## Author contributions

MP, DC, and LC contributed to conception of the work. LC wrote the manuscript. MP contributed to analysis of literature and table preparation. DC provided critical reading of the manuscript. All authors contributed to manuscript revision and approved the submitted version.
